# Uncovering Fahr’s Disease Through Psychotic Symptoms: A case report

**DOI:** 10.1192/j.eurpsy.2025.1673

**Published:** 2025-08-26

**Authors:** J. Zaouali, I. Dridi, S. Ellini, M. Cheour, W. Cherif, S. Hallit, F. Fekih-Romdhane

**Affiliations:** 1 Psychiatry, Razi Hospital, Tunis, Tunisia; 2 Research, School of Medicine and Medical Sciences, Holy Spirit University of Kaslik, P.O. Box 446, Jounieh, Lebanon; 3 Psychiatry, Tunis El manar university, faculty of Medicine of Tunis, Tunis, Tunisia

## Abstract

**Introduction:**

Bilateral pallidodentate calcinosis (BPC), also known as Fahr’s disease, is an uncommon neurodegenerative disorder that may be either genetic or sporadic. It is distinguished by bilateral and symmetrical deposits of calcium in the basal ganglia and occasionally in the cerebral cortex, impacting the regulation of motor and cognitive functions.

**Objectives:**

To underline the diagnostic pitfalls faced in Fahr’s disease when psychotic manifestations are predominant, thereby emphasizing the need to integrate neuroimaging and diagnostic assessments in differenciating this disease from a primary psychotic disorder.

**Methods:**

We present a rare case of Fahr’s disease with an atypical initial presentation, where the condition was initially misdiagnosed due to predominant psychiatric symptoms.

**Results:**

The patient, a 34-year-old man, displayed significant behavioral disturbances and cognitive decline since the age of 30. Initial psychiatric assessments identified a delusional syndrome, hallucinatory episodes, and psychomotor agitation, leading to a provisional diagnosis of a primary psychotic disorder. However, neuroimaging subsequently revealed bipallidal calcifications characteristic of Fahr’s disease, and further diagnostic evaluations (neurological examinations, a determination of parathyroid hormone (PTH) levels along with a comprehensive calcium-phosphate evaluation) confirmed the condition.The patient was treated symptomatically with second-generation antipsychotics, alongside supportive therapy, resulting in partial symptom alleviation.

**Conclusions:**

This case underscores the challenges of diagnosing Fahr’s disease when psychiatric manifestations predominate, which can delay appropriate diagnosis and treatment. Continuous, multidisciplinary follow-up is essential for optimal management of psychiatric symptoms.

**Disclosure of Interest:**

None Declared

